# Comparisons of Landscape Preferences through Three Different Perceptual Approaches

**DOI:** 10.3390/ijerph16234754

**Published:** 2019-11-27

**Authors:** Tian Gao, Huiyi Liang, Yuxuan Chen, Ling Qiu

**Affiliations:** College of Landscape Architecture and Arts, Northwest A&F University, Xianyang 712100, Shaanxi, China; tian.gao@nwsuaf.edu.cn (T.G.); lianghuiyi@nwafu.edu.cn (H.L.); cyx0724@nwafu.edu.cn (Y.C.)

**Keywords:** landscape perception and preference, on-site, photo, virtual reality

## Abstract

In order to identify the effects and divergences of the different landscape perception approaches on landscape preference, this study investigated people’s preferences for urban green spaces with different vegetation structures in the early spring through using three approaches, which were on-site survey, photo elicitation and VR technology. The results showed that: (a) There were significant differences among the three approaches for landscape preference, among which there was a significant difference between VR technology and the other two approaches, while no differences between on-site survey and photo elicitation were found. (b) The respondents showed significant differences in their preferences for the urban green spaces with the different vegetation structures through VR technology, and the semi-open green space received the highest preference score. (c) Whatever the approach employed, there were no significant differences in gender and professional background groups for landscape preference. (d) In the comparisons of the three different approaches, the respondents were more willing to choose physical recreational activities to be conducted in the early spring. Based on the above results, the three approaches of landscape perception were divergent and irreplaceable. It is, thus, suggested that the approach of landscape perception should be carefully selected for a specific landscape in a certain season, so as to provide a scientific basis for the evaluation of landscape perception and preference in the future.

## 1. Introduction

With the increase of urban population and the acceleration of urbanization, the urban landscape has changed rapidly. People’s perceptions of and preferences for landscape have correspondingly changed as well, which has inspired a greater number of scholars to conduct the study of landscape assessment from the public perspective [1]. However, even for a particular landscape or specific landscape elements, no unified conclusion of landscape preference has been reached at present due to the fact that different approaches have been adopted by various studies, which have been deemed controversial in equivalence and usefulness [2,3].

Previous studies on landscape preference generally emphasized the public perception of and preference for the landscape from the visual perceived sensory dimension. However, the approaches of visual landscape presentation have emerged endlessly to be applied to preference studies with different types of landscape; these approaches include on-site survey, photo elicitation, videos and virtual reality (VR) technology [1].

Since an on-site survey cannot only fully reflect site information and provide participants with the most authentic site experiences, but does maximize the simulation of their touring process, traditional preference studies are usually combined with questionnaires [4,5], interviews [6] and visitor employed photography (VEP) [7,8,9]. Nevertheless, an on-site survey often requires a large amount of manpower, material resources, financial resources and time, and coupled with some uncontrollable environmental and human interference factors, its effectiveness for obtaining useful and reliable information could be challenging.

For that reason and with the development of photo image technology, more and more researchers have used photo elicitation as the visual landscape presentation mode due to its strong manipulability, modifiability and easy accessibility. Participants can perceive different landscapes by means of photos, slides or websites, and finally, complete preference examinations by interviews [10,11,12], questionnaires [10,13,14,15], semantic difference scales [2,16] and oral questions [17]. Due to the limitations of the photography, the participants cannot comprehensively perceive and experience the site and capture the elements through other sensory reactions. The validity of preference could be influenced accordingly.

VR technology is an advanced technology combining a high degree of control and ecological validity, which can simulate experimental conditions that are reasonably similar to those in a real-world setting [18]. At present, there are two stimuli-presenting modes. One is to model and compose the scene using pictures through a variety of landscape elements, using computer-aided design (CAD), Geographic Information System (GIS) and other software, and then to construct a static or dynamic virtual digital landscape [19,20,21,22]. The other is to connect landscape materials collected on the sites or artificially synthesized materials (such as panoramic photos and venue recordings) to VR headsets, such as a VR headphone and VR earphone, so that participants can immerse themselves in the virtual world through VR devices, expecting to have the same experiences as the real landscape [23,24,25]. The key to the accuracy of VR technology lies in the realization of “presence”, which elicits the feeling of physically “being there” through the virtual environment [26], so that the virtual environment can reproduce users’ experiences in the real environment [27,28]. After all, since VR technology is a relatively new product to be used in landscape perception studies, it is quite necessary to verify whether VR technology can provide participants with a high enough degree of immersion and whether it can be used as an alternative to the experience of a real scene.

Although there are diversified approaches employed in the studies of landscape perception and preference, several questions arise, such as: Are the approaches being applied the same? Are they replaceable for collecting the information that regards perception and preference?

For photo elicitation and on-site surveys, some studies have shown that photos can reflect the real landscape well [29,30,31], while some studies were not consistent with this conclusion [32,33]. Al-Akl et al. found that the perception of the spiritual places with special significance (e.g., cemeteries), was limited to visual sense by the use of photo elicitation [10]. Gyllin and Grahn claimed that compared with lay persons, experts can more easily and deeply make sub-symbolic assessments of biodiversity on the site [2]. Sun et al. showed that the attentions of the participants varied for the same site when viewing the real scenes and seeing the photos [34].

Similarly, the relationship between VR technology and on-site survey has not yet been settled. Some studies have demonstrated that the immersive experience of VR technology is good and can be used as a substitute approach for physically experiencing the real scene [3]. Some studies even considered that VR could enhance the subjective experience of the environment as well as psychological and physiological reactions [35]. Chamilothori et al. found that there were no significant differences between a virtual environment and the real environment on the perceptions of subjects in the daylight [23]. Wei et al. showed that the visitors’ sense of presence in a VR environment has a positive impact on the experiences of a theme park and their willingness to revisit and provide recommendations to others [25]. However, Kjellgren and Buhrkall found that although both a real natural environment and simulated natural environment can reduce human stress, the former provided a greater and significant improvement of vigor and consciousness [36]. Therefore, the question whether VR can replace on-site survey for perception still remains to be examined. Since a systematically comparative study on the relationships among the on-site survey, photo elicitation and VR technology was lacking, this study was conducted to explore the similarities and divergences of the preferences for different landscapes through the use of three approaches in order to provide a scientific basis for the evaluation of landscape perception and preference in the future.

Due to the complexity of the term landscape, it is necessary to define a clear meaning for the landscapes that were studied in this study. A landscape mentioned in this paper refers to common, urban green spaces of small scale, and based on plants and perceptions and preferences, were examined correspondingly. In previous studies, not only the validity of perception approaches remained to be determined, but the comparison of perceptions of and preferences for different types of urban green spaces was lacking. Moreover, vegetation types (grass and trees) could differently affect perceptions and preferences, which could further influence people’s physiological and psychological well-being [37]. Hence, we chose different green spaces ranging from open to closed based on vegetation structure, which is an obvious visual feature to recognize different plant-based green spaces, aiming to find the differences among different types of urban green spaces. In addition, since the activities people conduct in the sites can reflect the effects of their perceptions and preferences to some extent, we also wanted to explore the activities people prefer to engage in. In particular, we aimed to investigate these questions:

1. Do the three approaches (on-site, photo elicitation and VR technology) have different effects on perceptions and preferences?
2. How do peoples’ preferences vary for different green spaces with different vegetation structures?
3. How do peoples’ preferences vary for activities in different spaces through different perceptual approaches?

## 2. Materials and Methods

### 2.1. Study Area

**Figure 1:**
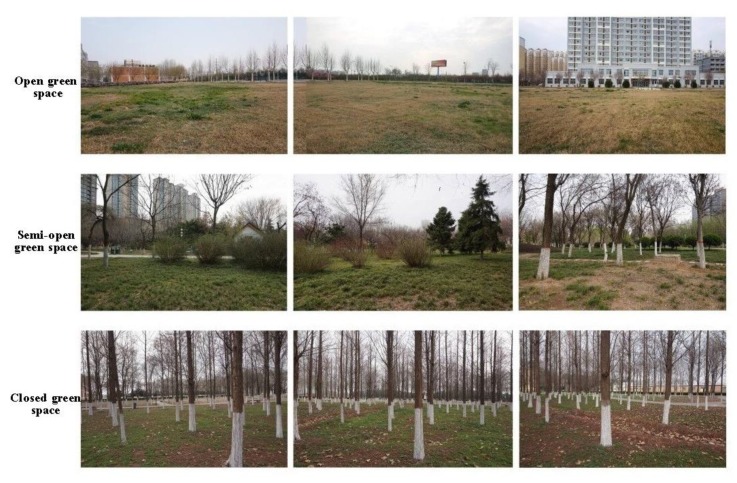
Two-dimensional color photos of the selected study areas.

Three types of urban green spaces were selected based on their similar size and the horizontal structure of vegetation according to the canopy cover ratio of trees and shrubs; the ratios were: open green space with less than 10% canopy of trees and shrubs, semi-open green space with a 30–70% canopy of trees and shrubs, and closed green space with more than 70% canopy of trees and shrubs (Figure 1).

### 2.2. Site Photos and VR Scenes

**Figure 2:**
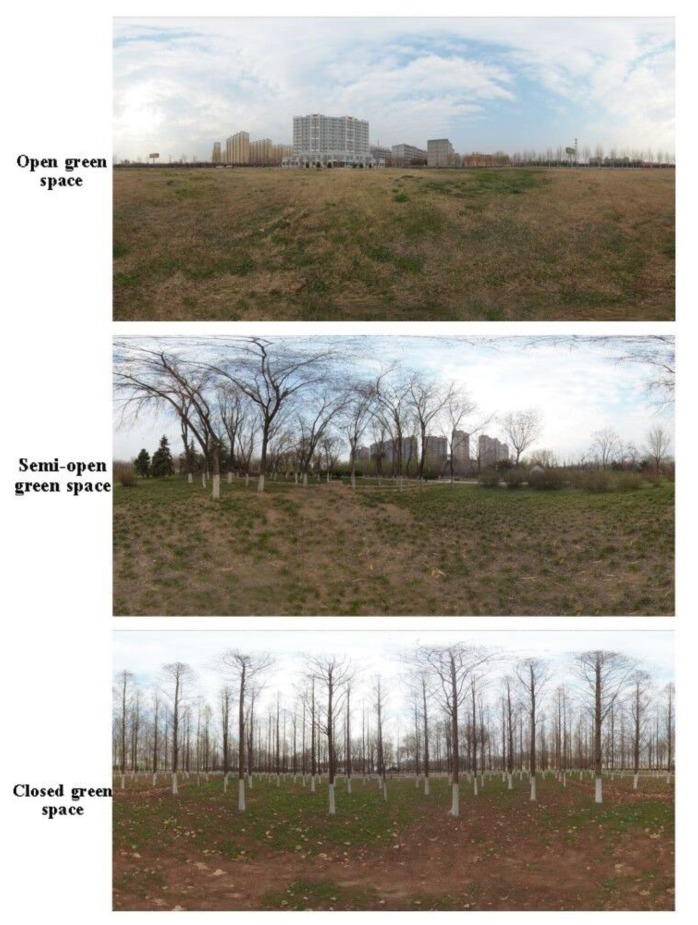
Panoramas photographs of the urban green spaces with the different horizontal structures.

This study was conducted around the spring equinox of the Chinese traditional solar terms in March 2019. First, two-dimensional color photos were taken by a digital camera (SONY ILCE-5000, Thailand) (Figure 1). In order to ensure that the photos contained all the scenes of each site, three photos were taken in each site with shooting angles of two photos 120° apart and the GPS coordinates of the shooting position were recorded as well. When the photographs were captured, the aperture, shutter and ISO were F4, 1/160 s and 200 respectively. Second, on the condition that visibility and light amount were roughly the same as those in 2D color photos, VR panoramic photos were shot by panoramic camera (Insta 360 Pro-I, Shenzhen, China) in RAW format at the same location according to the recorded GPS coordinates (Figure 2). The resolution of panoramic photos was 7680*3840 (8K) pixels. The aperture, shutter and ISO of the panoramic photos were F2.4, 1/2481 s and 100 respectively. Finally, three panoramic photos were displayed by VR glasses (Pico Goblin VR all-in-one, Beijing, China) with a resolution of 2560*1440 pixels to represent the selected three types of urban green spaces.

### 2.3. Respondents

**Figure 3:**
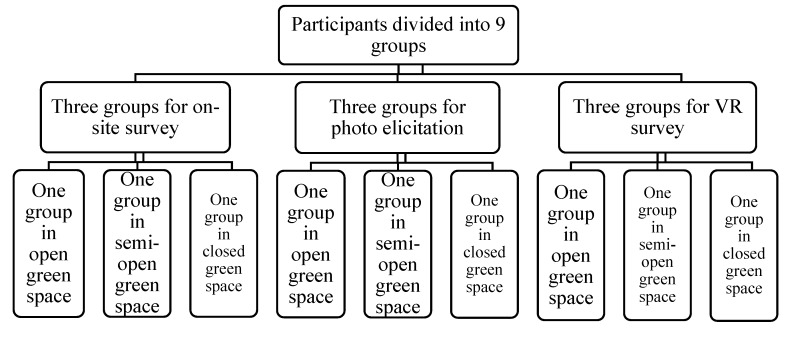
Group tasks arrangement of participants.

A total of 180 college students studying various subjects were recruited as the participants in this study and were divided into a professional group who studied the subject of landscape architecture and a non-professional group which included students who studied other subjects. The ratio of males to females was 1:1.5 and the ratio of professionals to non-professionals was 3:7. The participants were randomly divided into 9 groups with 20 people in each group, and every participant in a group was asked to use one approach to experience one type of urban green space (Figure 3).

### 2.4. Survey of Perception and Preference

In this study, a questionnaire survey was used to identify the participants’ perceptions of and preferences for the selected urban green spaces. The questionnaire consisted of three parts, including basic personal information (gender and profession), preferences and potential engagement in recreational activities. Preference was measured using a five-point Likert scale with the score of 1 to 5 representing “strongly dislike to strongly like”. In order to identify what kind of recreational activity people enjoyed through three perceptual approaches, recreational activities were divided into three categories: physical (playing ball games, flying kites, practicing martial arts and walking), social (picnicking, gatherings, chatting and playing games) and mental (emptying, meditating, sitting quietly and thinking). Before the beginning of the experiment, participants were provided an explanation of the purpose and the experimental process of this study.

**Figure 4:**
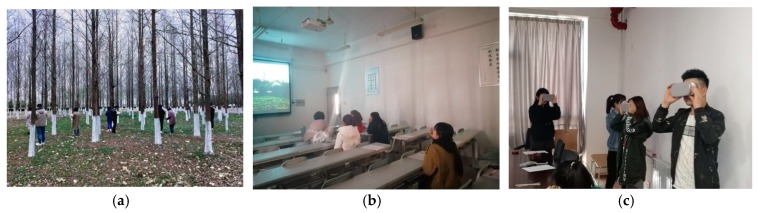
(**a**) Green space perception by on-site survey; (**b**) green space perception by photo elicitation; (**c**) green space perception by VR devices.

Three randomly chosen groups were each taken to one of the three different urban green spaces respectively for perceptions (Figure 4a). In order to avoid human interference, the participants in each group entered the site, and only 10 participants were allowed to stay on a site at once. The participants were allowed to walk freely to experience the site but were not allowed to communicate with others, and finally, each participant was asked to complete the questionnaire.

The off-site surveys were conducted in quiet and well-lit classrooms and conference rooms respectively. Another set of three groups perceived the three different urban green spaces through a slide projection (Figure 4b). Each photo of one green space type was displayed twice for 20 s in a loop. After all the presentations of photos of each green space type were finished, the participants were asked to complete the questionnaires. The last three groups were permitted to walk around freely to experience the selected green spaces through VR headsets, and then were asked to complete the questionnaire after 2 min of an immersion experience in the selected scene ensuring participants were granted a full experience of the environment (Figure 4c). A total of 179 valid questionnaires were collected because one participant was unable to engage in the on-site survey on time.

### 2.5. DataAnalysis

Participants in different gender and professional background groups were not evenly distributed between the sites, and accordingly a generalized linear model (GLM) was applied to the whole sample to identify the effects of most decisive factors (gender, professional background, type of green space and approach) on preference. To identify the particular differences among different approaches and different green spaces, the post-hoc tests using Duncan’s *t*-test, were then analyzed to compare the different levels of preference among the three approaches and three green spaces respectively. To determine the significant differences among three approaches emerged in which spaces, the posterior comparisons of different approaches by Duncan’s *t*-test in each green space, were also conducted. To identify the possible reasons and the detailed aspects of respondents’ preferences, the willingness to visit and potential recreational activities were analyzed by descriptive statistics. All statistical analyses were performed using SAS (version 9.4, Statistical Analysis System, US) software. Qualitative analysis was finally conducted to identify the similarities and divergences of the three approaches on preference for the selected green spaces based on the comments of the participants.

## 3. Results and Discussion

### 3.1. Factors Affecting Landscape Preference

The results of generalized linear model were showed in Table 1. The model showed that approach and type of green space had significant influence on preference (*p* < 0.05), while gender and professional background had no influence (*p* > 0.05, Table 1b). This indicated that the participants had significant differences of preference for the urban green spaces through the three approaches. 

In the present study, gender and profession had no influence on the preference. The result is consistent with the findings of Lyons [38], but in opposition to the findings of other studies [17,39,40]. According to Lyons, the preferences of people of different genders may be associated with age and residence, while the respondents in this study were all college students with similar age, living at school [38]. What is more, although the selected sites in this study were different in vegetation structure, they were all common and routine spatial types in urban environments and were familiar to students. For the spaces having little novelty or distinctive features, the influence of gender and profession on the preferences was not significant. Thus, in order to identify the effects of gender and professional background on preference for green spaces, appropriate survey site selection is quite important in further research.

#### 3.1.1. Differences in Landscape Preference among Different Perception Approaches

To compare the difference among three approaches, the post-hoc test was conducted and results showed that there were significant differences between VR technology and the other two approaches, but no differences between photo elicitations and on-site survey (Table 2). VR technology got the highest preference scores, indicating that participants preferred the virtual environment best, far more than photos and real scenes.

It indicated that there were differences in perception of the selected green spaces among approaches and VR cannot replace the other two perception approaches, while photos seemed to be a good representation of real environments. The difference between VR technology and on-site may be due to the different senses of presence in virtual environments from reality. In a virtual context, presence is commonly defined as the subjective experience of being there in the technology-mediated environment [41,42]. Hence, people should generate the same perceptions in virtual environments as in reality if they obtain sufficient feelings of presence in virtual environments as there are in reality. The experience of presence is a complex, multidimensional perception, which is formed through an interplay of multi-sensory information and various cognitive processes [28]. Therefore, in this study, participants using VR devices could not produce different multi-sensory outputs indoors that are present outdoors. In the process of green spaces perception, due to the deficiency of other sensory process, some attributes, such as sound, air humidity, temperature, wind, and ground texture, etc., cannot be reflected in virtual environments, which limit the sensory activity to the vision, and that influences the feeling of presence.

On the other hand, Schuemie et al. claimed that vital to presence is the suppression of information that is incompatible with the VR experience [43]. That means that only if a virtual scene conforms to people’s presuppositions and cognition of the scene in reality, can it generate a similar presence to reality. In the present study, when participants were immersed in a virtual environment, the objects (like leaves, grass) were stationary and at a constant distance with people, even if people tried to walk closed to them, which was not accordance with real circumstances. In addition, in the virtual experience, the appearance of the VR device and the direct or indirect attributes of product, such as VR display equipment itself, visual resolution, users’ different sensory perceptions and even adverse physiological reactions to VR devices could influence users’ multi-senses to some extent [44,45]. Thus, it is indicated that the incompatible factors in virtual environments and experience affect the presence, further arousing different effects of perceptions.

No significant difference emerged between the on-site approach and photo elicitation, indicating that photographs could be used as a substitute for on-site perception, consistent with some previous studies. Coeterier showed that photographs could be substituted for reality for some scenes, such as small-scale landscapes, but not large-scale landscapes with micro-relief [46]. In this study, the selected green spaces were common small-scale urban landscapes so it was undemanding for participants to recognize and perceive the spaces. Moreover, Cortignani et al. mentioned that some interviewees would perceive environmental benefits in addition to visual perception of the rural landscape through photographs [47], indicating that to some extent photographs would not limit the landscape perception. In our study, the three spaces were common and familiar to respondents; thus, in addition to visual sense, photographs may have aroused respondents’ other experiences and cognition similar to reality.

The result that participants had highest preferences for VR technology indicated that compared with the other two approaches, VR experience contributed to producing more positive experiences in the green spaces. This is consistent with the previous study of Wei et al., who claimed that visitors’ greater satisfaction with the theme park as well as their increased intentions to revisit and recommend the park to others was due to visitors’ sense of presence in a virtual environment, and the presence predominantly resulted from the particular experiential attributes (feeling of control, participation and curiosity) of VR technology application [25]. Therefore, the VR device is not only the carrier of visual landscape, but also a relatively new technological product. The participants’ strongest preferences could be due to the novel experience of application of VR devices.

The posterior comparisons among the different approaches in each type of green space showed that VR had the highest preference scores among all the types of the selected green spaces (Table 3). Participants only had significantly different perceptions of and preferences for the semi-open green spaces in regard to the approaches, of which there was a significant difference between photo elicitation and VR technology. One possible explanation for the lack of difference in application of the three approaches is that compared with the semi-open green space with multi-layer structure, open and closed green spaces had simple structures with scarce species and a relatively monotonous environment. Sun et al. found that more elements were noticed when viewing a scene through photo elicitation, while fewer were through on-site surveys where participants spent a longer time viewing the main elements [34]. In photo elicitation, the feature that people notice more elements but have short fixation would be associated with a less engaged relationship to the space; inversely, the more time people fix on main elements on site, the more positive the evaluation of the space. Although a feature of elements captured in virtual environments has not been found, it is possible that the number of elements focused on and the duration of focus could be the source of the difference among three approaches, which would be obvious in complex landscapes like the semi-open green space. Therefore, the richness of landscape elements may be the most common causes of divergence in application of the various perception approaches, which should be taken into account when selecting perception approaches.

Further analysis of the comments of the semi-open green space showed that nearly half of the participants perceived the tranquility by using VR devices, while noise was experienced by more than a quarter of the participants on the site. Since the selected site was a park where inhabitants often get together to play instruments, sing and chat, the noisier environment could be the reason for the perceptive differences between on-site and off-site approaches.

#### 3.1.2. Participants’ Preferences for Different Types of Green Spaces

To compare the preferences for different urban green spaces with different structures, the post-hoc test was conducted and the results shown in Table 4 show a significant difference between preference scores of semi-open space and open space, with the semi-open space receiving the highest preference score, which was followed by closed space; last was open space. According to the questionnaires, the main reason why semi-open space attracted the participants was due to the beautiful plants and comfortable and relaxing atmosphere. The most negative responses received for the open space from the participants were the vegetation (“mottled grassland”, “poor greening”, “few tree species”), followed by the atmosphere of the site (“empty and cold”, “desolate”, “single scenery”). For the closed green space, participants considered that this was a gloomy environment with dense and single trees and no recreational facilities but had better privacy and shade.

The attributes of the semi-open environment that attracted participants may concern two aspects: the moderate degree of openness, which means both a certain extent of openness and a certain degree of shade, and the moderate vegetation density along with plant diversity. The former factor may be explained by the prospect-refuge theory, which showed that the ability to see without being seen was important to meet early man’s survival needs [48], and the attributes of an environment that ensure satisfaction of survival needs can become sources of aesthetic satisfaction and preference [49]. In response to this theory, the semi-open space could meet the participants’ needs of both looking out and feeling safe. Another reason was consistent with the findings of previous studies [5,15]. There is a positive relationship between aesthetics and biodiversity which can be improved by the addition of trees [50], but note that too densely-packed trees will limit vision and reduce the sense of security [51]. So an appropriate degree of vegetation can contribute a lot to preference in environments. In addition, since the study was conducted in early spring, the plant landscape in the semi-open space was better than the other environments for there were more deciduous trees in the former environment. Several studies showed that the visual characteristics of plants in different seasons can influence the perception of landscape preference criteria [52]. This indicates that season is an important factor in the study of landscape preference, which needs to be taken into consideration in future studies.

### 3.2. Activity Preferences

**Figure 5:**
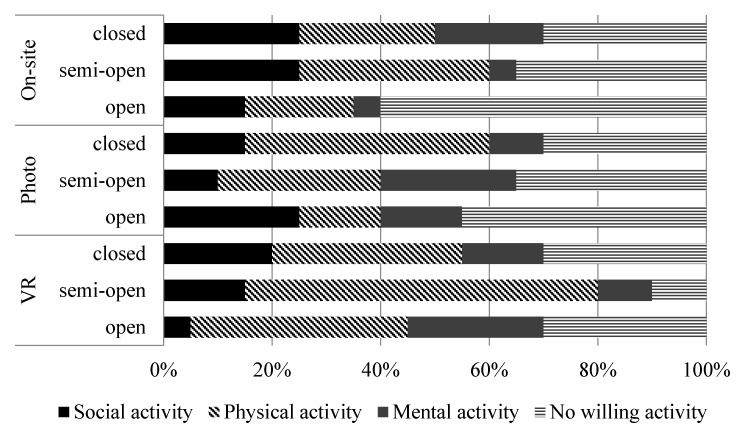
Activities selected by participants in different green spaces.

Among the 179 questionnaires, 121 participants were willing to engage in a variety of activities in early spring. The highest frequency of the activities was reported for walking (45.5%), which was followed by picnicking (9.9%) and chatting (9.9%).The results showed that physical activities were the most popular categories that participants were willing to engage in, followed by social activities, while mental activities were the least popular (Figure 5). By realizing the differences of activities in three types of green spaces, it was concluded again that three approaches were irreplaceable.

Participants in the VR technology group were more likely than those in other groups to engage in physical activities (Figure 5). Quietness was considered to be one of the main reasons for people to participate in activities according to the comments. It could be explained by psychological stress relief with the contribution from VR devices not reflecting the sounds in green spaces, because audio input in a mixed audio-visual assessment of environmental representations could have a stronger influence than visual input [53].

Participants in the photo elicitation group preferred social activities in open green space, while they preferred physical activities in semi-open and closed green spaces (Figure 5). Green space was considered wild and peaceful, which may also have been affected by the lack of sound. Moreover, in terms of the total three activities, the number of participants in the photo elicitation group willing to engage in activities was fewer than the VR technology group, which may be attributed to the fact that VR devices were not only more attractive than photos but also more easily able to arouse peoples’ interest in entering green space.

Participants in the on-site survey group preferred physical activities in semi-open green space, while the number of social activities chosen was nearly as large as the number of physical activities in open and closed green spaces (Figure 5). In open green space, participants would tend to fly kites instead of walking because of the wideness of the site and nice weather. However, fewer participants were willing to engage in activities in the on-site survey group. Some participants thought that the environment was noisy, the temperature was unsuitable and the infrastructure needed maintenance. These factors cannot be embodied in photos and VR devices.

## 4. Conclusions

This study was the first to explore the divergences of preferences for different urban green spaces using three approaches and aimed to provide a basis of knowledge for the evaluation of landscape perception and preference. There were some noteworthy conclusions. First, VR technology significantly differed from the other two approaches, especially for the semi-open green space, while no difference between on-site survey and photo elicitation was found. Second, there was a significant difference between preferences for green spaces with different vegetation structure in early spring. Semi-open green space received the highest preference scores. That was followed by closed green space, and open green space was rated the lowest. Finally, physical activities were the most popular activity categories that participants were willing to engage in during the early spring. Based on these results, it is suggested that appropriate approaches should be selected according to the sites’ actual environments, especially for VR technology, which would be cautiously utilized for sites with strong heterogeneity, complex elements and diverse spatial structures of vegetation.

However, some weaknesses in this study exist. First, VR technology was only used to provide visual scenes during this study. Since landscape perception is a multi-sensory process, further research could utilize multiple VR devices (a VR helmet and VR headset) to better present the scenery in all sensory dimensions. Second, all participants were college students, so the results might not reflect other social groups. Other varieties of samples should be further examined. Third, the spaces selected in this study were common for the participants. In order to fully explore the divergences in age and professional groups, other types of landscapes, such as natural or semi-natural sites far away from urban areas should be taken into account. Finally, since this study was only conducted in early spring, the participants’ preferences may have been greatly affected by the erratic weather and seasonal plant aspects, especially through on-site survey approach. Further research could be conducted in multiple seasons for comparison.

## Figures and Tables

**Table:** **a.** The overall information about the generalized linear model (GLM) model.

	df	Sum of Squares	Mean Square	F Value	Pr > F
GLM Model	6	15.74	2.62	4.22	0.00
Error	172	106.94	0.62		
Corrected Total	178	122.68			

**Table:** **b.** The results of type Ⅲ Sum of Squares (SS) and type I Sum of Squares (SS).

	df	III-SS	Mean Square	F Value	Pr > F	I-SS	Mean Square	F Value	Pr > F
Gender	1	2.49	2.19	3.52	0.06	3.58	3.58	5.75	0.02
Subject	1	1.46	1.46	2.35	0.13	3.41	3.41	5.49	0.02
Type of green space	2	4.22	2.11	3.39	0.04	4.15	2.07	3.34	0.04
Approach	2	4.60	2.30	3.70	0.03	4.60	2.30	3.70	0.03

**Table 2:** Posterior comparisons of different approaches.

Approach	Mean	*N*	Duncan Group
On-site survey	3.32	59	B
Photo elicitation	3.37	60	B
VR technology	3.75	60	A

**Table 3:** Posterior comparisons of different approaches in each green space.

Approach	Open Green Space		Semi-Open Green Space		Closed Green Space	
	Mean	Duncan Group	Mean	Duncan Group	Mean	Duncan Group
On-site survey	3.00	A	3.58	BA	3.55	A
Photo elicitation	3.25	A	3.35	B	3.35	A
VR technology	3.55	A	4.05	A	3.65	A

**Table 4:** Posterior comparisons of landscape types.

Green Space	Mean	*N*	Duncan Group
Semi-open space	3.66	59	A
Closed space	3.52	60	B A
Open space	3.27	60	B

## References

[1] Qi T., Wang Y.J., Wang W. (2013). A review on visual landscape study in foreign countries. Prog. Geogr..

[2] Gyllin M., Grahn P. (2015). Semantic Assessments of Experienced Biodiversity from Photographs and On-Site Observations—A Comparison. Environ. Nat. Resour. Res..

[3] Yu C., Lee H., Luo X. (2018). The effect of virtual reality forest and urban environments on physiological and psychological responses. Urban For. Urban Green..

[4] Oguz D., Dirioz E.D., Belkayali N. (2010). Tourists’ perception of landscape design: The case of resorts in the Belek Specially Protected Area. Afr. J. Agric. Res..

[5] Zhang H., Chen B., Sun Z., Bao Z. (2013). Landscape perception and recreation needs in urban green space in Fuyang, Hangzhou, China. Urban For. Urban Green..

[6] Muratet A., Pellegrini P., Dufour A., Arrif T., Chiron F. (2015). Perception and knowledge of plant diversity among urban park users. Landsc. Urban Plan..

[7] Gou S., Shibata S. (2017). Using visitor-employed photography to study the visitor experience on a pilgrimage route—A case study of the Nakahechi Route on the Kumano Kodo pilgrimage network in Japan. J. Outdoor Recreat. Tour..

[8] Nielsen A.B., Heyman E., Richnau G. (2012). Liked, disliked and unseen forest attributes: Relation to modes of viewing and cognitive constructs. J. Environ. Manag..

[9] Qiu L., Lindberg S., Nielsen A.B. (2013). Is biodiversity attractive?—On-site perception of recreational and biodiversity values in urban green space. Landsc. Urban Plan..

[10] Al-Akl N.M., Karaan E.N., Al-Zein M.S., Assaad S. (2018). The landscape of urban cemeteries in Beirut: Perceptions and preferences. Urban For. Urban Green..

[11] Fyhri A., Jacobsen J.K.S., Tømmervik H. (2009). Tourists’ landscape perceptions and preferences in a Scandinavian coastal region. Landsc. Urban Plan..

[12] Larsen L., Harlan S.L. (2006). Desert dreamscapes: Residential landscape preference and behavior. Landsc. Urban Plan..

[13] Kalivoda O., Vojar J., Skřivanová Z., Zahradník D. (2014). Consensus in landscape preference judgments: The effects of landscape visual aesthetic quality and respondents’ characteristics. J. Environ. Manag..

[14] López-Martínez F. (2017). Visual landscape preferences in Mediterranean areas and their socio-demographic influences. Ecol. Eng..

[15] Wang R., Zhao J., Meitner M.J., Hu Y., Xu X. (2019). Characteristics of urban green spaces in relation to aesthetic preference and stress recovery. Urban For. Urban Green..

[16] Natori Y., Chenoweth R. (2008). Differences in rural landscape perceptions and preferences between farmers and naturalists. J. Environ. Psychol..

[17] Zheng B., Zhang Y., Chen J. (2011). Preference to home landscape: Wildness or neatness?. Landsc. Urban Plan..

[18] Bohil C.J., Alicea B., Biocca F.A. (2011). Virtual reality in neuroscience research and therapy. Nat. Rev. Neurosci..

[19] Fisher-Gewirtzman D. (2018). Perception of density by pedestrians on urban paths: An experiment in virtual reality. J. Urban Des..

[20] Griffon S., Nespoulous A., Cheylan J., Marty P., Auclair D. (2011). Virtual reality for cultural landscape visualization. Virtual Real. Lond..

[21] Lange E. (2001). The limits of realism: Perceptions of virtual landscapes. Landsc. Urban Plan..

[22] Lin C., Thomson G., Hung S., Lin Y. (2012). A GIS-based protocol for the simulation and evaluation of realistic 3-D thinning scenarios in recreational forest management. J. Environ. Manag..

[23] Chamilothori K., Wienold J., Andersen M. (2019). Adequacy of Immersive Virtual Reality for the Perception of Daylit Spaces: Comparison of Real and Virtual Environments. Leukos.

[24] Jung T., Dieck M.C.T., Moorhouse N., Dieck D.T. Tourists’ Experience of Virtual Reality Applications. Proceedings of the 2017 IEEE International Conference on Consumer Electronics (ICCE).

[25] Wei W., Qi R., Zhang L. (2019). Effects of virtual reality on theme park visitors’ experience and behaviors: A presence perspective. Tour. Manag..

[26] Slater M., Wilbur S.A. (1997). Framework for Immersive Virtual Environments (FIVE): Speculations on the Role of Presence in Virtual Environments. Presence Teleoper. Virtual Environ..

[27] de Kort Y., Jolien W.A.I., Kooijman J., Schuurmans Y. (2003). Virtual Laboratories: Comparability of Real and Virtual Environments for Environmental Psychology. Presence Teleoper. Virtual Environ..

[28] Diemer J., Alpers G.W., Peperkorn H.M., Shiban Y., Mühlberger A. (2015). The impact of perception and presence on emotional reactions: A review of research in virtual reality. Front. Psychol..

[29] Iv R.B.H., Stewart W. (1992). Validity of photo-based scenic beauty judgments. J. Environ. Psychol..

[30] Nassauer J.I. (1983). Framing the Landscape in Photographic Simulation. J. Environ. Manag..

[31] Stewart T.R., Downton P.M.M., Ely D. (1984). Judgments of photographs vs. field observations in studies of perception and judgment of the visual environment. J. Environ. Psychol..

[32] Gibsen J.J. (1979). The Ecological Approach to Visual Perception.

[33] Valtchanov D., Ellard C.G. (2015). Cognitive and affective responses to natural scenes: Effects of low level visual properties on preference, cognitive load and eye-movements. J. Environ. Psychol..

[34] Sun M., Herrup K., Shi B., Hamano Y., Liu C., Goto S. (2018). Changes in visual interaction: Viewing a Japanese garden directly, through glass or as a projected image. J. Environ. Psychol..

[35] Simon S.C., Greitemeyer T. (2019). The impact of immersion on the perception of pornography: A virtual reality study. Comput. Hum. Behav..

[36] Kjellgren A., Buhrkall H. (2010). A comparison of the restorative effect of a natural environment with that of a simulated natural environment. J. Environ. Psychol..

[37] Huang Q., Yang M., Jane H., Li S., Bauer N. (2020). Trees, grass, or concrete? The effects of different types of environments on stress reduction. Landsc. Urban Plan..

[38] Lyons E. (1983). Demographic Correlates of Landscape Preference. Environ. Behav..

[39] Wang R., Zhao J. (2017). Demographic groups’ differences in visual preference for vegetated landscapes in urban green space. Sust. Cities Soc..

[40] Wang R., Zhao J., Meitner M.J. (2017). Urban woodland understory characteristics in relation to aesthetic and recreational preference. Urban For. Urban Green..

[41] Slater M. (1999). Measuring presence: A response to the Witmer and Singer presence questionnaire. Presence.

[42] Steuer J. (1992). Defning virtual reality: Dimensions determining telepresence. J. Commun..

[43] Schuemie M., Van Der Straaten P., Krijn M., Van Der Mast C.A.P.G. (2001). Research on presence in virtual reality: A survey. Cyber Psychol. Behav..

[44] Bystrom K.E., Barfield W., Hendrix C.M. (1999). A Conceptual Model of the Sense of Presence in Virtual Environments. Presence Teleoper. Virtual Environ..

[45] Van der Spek E.D., Houtkamp J.M. Mixing Emotions, How Physical Discomfort Influences the Affective Appraisal of Virtual Places. Proceedings of the 2008 International Conference Visualisation.

[46] Coeterier J.F. (1983). A photo validity test. J. Environ. Psychol..

[47] Cortignani R., Gobattoni F., Pelorosso R., Ripa M.N. (2018). Green Payment and Perceived Rural Landscape Quality: A Cost-Benefit Analysis in Central Italy. Sustainability.

[48] Appleton J. (1975). The Experience of Place.

[49] Balling J.D., Falk J.H. (1982). Development of Visual Preference for Natural Environments. Environ. Behav..

[50] Parsons R., Daniel T.C. (2002). Good looking: In defense of scenic landscape aesthetics. Landsc. Urban Plan..

[51] Laing R., Davies A., Miller D., Conniff A., Scott S., Morrice J. (2009). The application of visual environmental economics in the study of public preference and urban green space. Environ. Plann. B.

[52] Kuper R. (2013). Here and Gone: The Visual Effects of Seasonal Changes in Plant and Vegetative Characteristics on Landscape Preference Criteria. Landsc. J..

[53] Preis A., Kocinski J., Hafke-Dys H., Wrzosek M. (2015). Audio-visual interactions in environment assessment. Sci. Total Environ..

